# Spontaneous Atopic Dermatitis-Like Symptoms in *a/a ma ft/ma ft/J* Flaky Tail Mice Appear Early after Birth

**DOI:** 10.1371/journal.pone.0067869

**Published:** 2013-07-03

**Authors:** Magdalini Kypriotou, Cloé Boéchat, Marcel Huber, Daniel Hohl

**Affiliations:** Laboratory of Cutaneous Biology, Service of Dermatology and Venereology, Beaumont Hospital CHUV, Lausanne, Switzerland; CNRS-University of Toulouse, France

## Abstract

Loss-of-function mutations in human profilaggrin gene have been identified as the cause of ichthyosis vulgaris (IV), and as a major predisposition factor for atopic dermatitis (AD). Similarly, flaky tail (*a/a ma ft/ma ft/J*) mice were described as a model for IV, and shown to be predisposed to eczema. The aim of this study was to correlate the flaky tail mouse phenotype with human IV and AD, in order to dissect early molecular events leading to atopic dermatitis in mice and men, suffering from filaggrin deficiency. Thus, 5-days old flaky tail pups were analyzed histologically, expression of cytokines was measured in skin and signaling pathways were investigated by protein analysis. Human biopsies of IV and AD patients were analyzed histologically and by real time PCR assays. Our data show acanthosis and hyperproliferation in flaky tail epidermis, associated with increased IL1β and thymic stromal lymphopoietin (TSLP) expression, and Th2-polarization. Consequently, NFκB and Stat pathways were activated, and IL6 mRNA levels were increased. Further, quantitative analysis of late epidermal differentiation markers revealed increased Small proline-rich protein 2A (Sprr2a) synthesis. Th2-polarization and Sprr2a increase may result from high TSLP expression, as shown after analysis of 5-days old K14-TSLP tg mouse skin biopsies. Our findings in the flaky tail mouse correlate with data obtained from patient biopsies of AD, but not IV. We propose that proinflammatory cytokines are responsible for acanthosis in flaky tail epidermis, and together with the Th2-derived cytokines lead to morphological changes. Accordingly, the *a/a ma ft/ma ft/J* mouse model can be used as an appropriate model to study early AD onset associated with profilaggrin deficiency.

## Introduction

The spontaneously occuring “*flaky tail*” mouse strain *a/a ma ft/ma ft/J* was originally described as a model of human IV (OMIM 146700) due to its reduced expression of profilaggrin and keratohyalin granules [1]. Accordingly, *a/a ma ft/ma ft/J* mice carry the recessively transmitted *flg* loss-of-function mutation 5303delA [2]. At birth, they appear normal, and the flaky phenotype becomes visible at 3 days after birth with the presence of dry scaly skin and tail constrictions. This phenotype reaches a peak at around day 6, then it resolves progressively; at day 21, the skin appears normal, but the mice remain smaller than wild type littermates, they have shorten ears and lack the tail tips [1], [3]. Following the identification of IV as the major risk factor for developing AD and asthma [4], several studies revealed an epidermal barrier disruption and the predisposition of “flaky tail” mice to develop eczema [2], [5], [6]. *a/a ma ft/ma ft/J* mice have increased TEWL and skin permeability and they are susceptible to develop allergic immune responses when challenged with ovalbumin or dust mites [2], [5], [6], [7], [8]. The “flaky tail*”* mouse strain not only carries *flg* mutations, but also the closely linked *matted* mutation on mouse chromosome 3 (*ma/ma*), which is responsible for abnormal hair coat [1].

Profilaggrin, the major component of keratohyalin granules, is encoded by the second and third exon of the *FLG* gene. During later differentiation, profilaggrin is dephosphorylated and processed by furin into the N-terminus and the rest of the protein. Multimeric filaggrin is processed into oligomeric, and then single filaggrin repeats [9]. The C-terminus is indispensable for the profilaggrin to filaggrin processing, because truncating mutations close to the C-terminus are sufficient to inhibit formation of filaggrin monomers [1], [10]. Filaggrin peptides aggregate the keratin cytoskeleton, cause collapse of the granular cells into flattened anuclear squames, and contribute to the formation of corneocytes [11]. In the stratum corneum (SC), caspase 14 and calpain 1 further degrade filaggrin units into free hydrophilic amino-acids or amino-acid byproducts [12], [13] which form the Natural Moisturizing Factor (NMF). NMF is crucial for the maintenance of hydration and pH levels of the upper epidermis, and, especially urocanic acid, for the skin protection from UV light [13].

Heterozygous *FLG* mutations cause a mild form of IV which affects about 10% of European population, whereas homozygous mutations lead to a more severe IV occuring in 1∶730 individuals [4]. IV is clinically characterized by palmar hyperlinearity, keratosis pilaris and dry skin with prominent scales on the lower abdomen, arms and legs [4]. Histologically, heterozygous and homozygous IV show reduced or absent keratohyalin granules, respectively, and retention orthokeratosis. *FLG* and therefore keratohyalin deficiency is strongly correlated with an early and persistent onset of AD, since 47% of IV patients suffer from it.

AD (OMIM 603165) is the most common dermatitis in children and predisposes to asthma and allergic rhinitis [14]. Histologically, AD is characterized by acanthosis, spongiosis, prominent Langerhans cells (LCs) and eosinophilia, monocyte-macrophage infiltrates and mast cells in the dermis [15]. Its onset involves an initial strong Th2-cell polarization induced either by external factors and/or by specific cytokines, such as TSLP, produced by resident cells [16].

In this study, we set out to better understand the pathophysiology of IV and the biological trail connecting IV with AD using *a/a ma ft/ma ft/J* mice. As a basis, we questioned whether the “flaky tail” reproduces better the human IV or/and AD phenotype, and what are the first molecular signs leading from impaired barrier to eczema. Five days old (P5) pups were used in order to witness early life consequences in epidermis function. Histological analysis revealed acanthosis and inflammatory infiltrates in the dermis, associated with increased IL1β and TSLP mRNA levels. IL1β upregulation was linked to NFκB activity and to increased IL6, VCAM and ICAM expression. Further, increase of Small proline-rich protein 2 (SPRR2) expression suggests potential compensation for filaggrin [17]. Real time PCR analysis of AD-patient biopsies showed increased IL1β, IL13 and SPRR2a mRNA expression which was not the case for IV-patients. These findings demonstrate that the phenotype of the *a/a ma ft/ma ft/J* mice reflects better human AD and open new directions of research on the consequences of defective barrier [5], [7], [18].

## Materials and Methods

### Mice

Homozygous flaky tail (*a/a ma ft/ma ft/J*) mice, kindly provided by Dr. P. Fallon (Dublin, Ireland), K14-TSLP tg mice, kindly provided by Prof. F. Radtke (EPFL, Lausanne, Switzerland) and wild type (WT) (C57blk6/J) (Charles River, France) mice were bred in SPF conditions according to federal guidelines and the federal and local authorities approved procedures (permit number: VD2356; VD2102). All biopsies used in this study derived from 5 days old pups (P5) euthanized by decapitation.

### Human

Human skin biopsies were collected from patients who came to our clinic for medical consulting (Dermatology Service, Beaumont Hospital, CHUV, Lausanne, Switzerland). The patients provided written and informed consent (Geneskin protocol approved 27.10.2007 by the local Ethical Committee).

Patients DNA was extracted from skin biopsies using a DNeasy Blood & Tissue Kit (Qiagen, Germany). Profilaggrin mutations were detected as described previously: R501X and 2282del4 [4]; R2447X mutation [19]. Detailed information about the patient biopsies can be found in Tables S1 in File S1.

### RNA Isolation and Real Time PCR

Total RNA from skin biopsies was extracted using the RNeasy Fibrous Tissue Mini Kit (Qiagen, Germany). RNA integrity was verified on an agarose gel under denaturating conditions. RNA (2 µg) was reverse-transcribed into cDNA using MMLV-reverse transcriptase (New England Biolabs, UK) as follows: 10 min at 25°C, 60 min at 42°C, 5 min at 95°C. Real-time PCR analysis was performed on a StepOneTM PCR apparatus (Applied Biosystems, UK) using a Power SYBR Green Master Mix (Applied Biosystems, UK) and specific primers. Samples were amplified as described [20]. Primers were designed using the Roche software (Universal Probe Library, Assay Design Center), unless described differently (Tables S2 and S3 in File S1). Analysis of relative gene expression was performed using the 2^−ΔΔCT^ method [21]. Hprt and Gapdh mRNA were used as internal controls for mouse samples and RPL13a for human skin.

### Immunohistochemistry

Dorsal biopsies were snap frozen in isopentane, then embedded in OCT and cut in 5 µm cryosections. Specimens were fixed in ice cold acetone, then blocked with 5% NGS (normal goat serum) – TBS – GBA (glycine – BSA) for 1 hr and incubated at RT with the primary antibody (Table S4 in File S1). 3 hrs later, slides were rinsed and incubated with a 488-Alexa Fluor secondary antibody (Molecular Probes, Netherlands) for 1 hr at RT. Counterstaining was realized with DAPI and mounting with a Fluorescent Mounting medium (Dako, Denmark). The images were captured by a confocal microscope (LSM 700 Zeiss, Switzerland) and analyzed using the ZEN2010 software.

### Western Blotting

Dorsal biopsies were submitted to a heat shock (1 min 60°C - 30 sec 4°C) in PBS to separate dermis from epidermis. Epidermis was pulverized in liquid nitrogen, then homogenized in extraction buffer: 150 mM NaCl, 50 mM Tris HCl (pH 8), 5 mM EDTA (pH 8), 1% Nonidet-P40, protein inhibitors (Complete mini, EDTA-free; PhosphoSTOP, Roche, Switzerland). The samples were centrifuged 5 min at 12000 rpm (4°C) and the supernatant containing the soluble protein fraction was separated. The pellet was incubated in the same buffer containing 9 M Urea –50 mM DTT for 1 hr at 4°C; the insoluble fraction was obtained after centrifugation (10 min, 12000 rpm, 4°C) [22]. Proteins were assayed by Bradford method. Lysates were fractionated on SDS-PAGE gels, electrotransferred onto PVDF Immobilion®-P Transfer membranes (Millipore, Temecula, CA) and stained with Ponceau S to evaluate protein loading. Membranes were blocked with TBST (20 mM Tris–HCl, pH 7.5, 150 mM NaCl, 0.2% Tween-20) plus 10% non-fat dry milk (NFDM) and incubated with primary antibodies (Table S4 in File S1) in TBST- 5% NFDM overnight at 4°C. Horseradish peroxidase conjugated IgG anti-mouse or anti-rabbit were used as secondary antibodies (GE Healthcare, UK), in TBST- 2% NFDM for 1 hr at RT. Antigen bands were revealed by chemiluminescence (ECL Plus Kit, GE Healthcare, UK) in a LAS4000 imaging system (Berthold Mithras, Switzerland). Normalization to β-actin and quantification was performed using Photoshop CS3.

### Histology

Skin biopsies were fixed overnight in fresh PFA 4% at 4°C, then washed in TBS and embedded in paraffin. Specimens of 5 µm thickness were stained with hematoxylin and eosin as described [20]. Stained sections were analyzed under light microscope (Nikon Ellipse E400, Switzerland) with the AxioVision software (Switzerland).

### Statistical Analysis

Experiments were analyzed with the 2-tailed Student t-test, using the GraphPad prism 6.0 software (GraphPad Software, Inc, San Diego, CA, USA). Results are presented as means ± SEM, unless otherwise described in the figure legends. P-values lower than 0.05 were considered statistically different.

## Results

### Hypercellularity, Acanthosis and Keratin 6 Expression in 5-days Old Flaky Tail Mouse Skin

**Figure 1:**
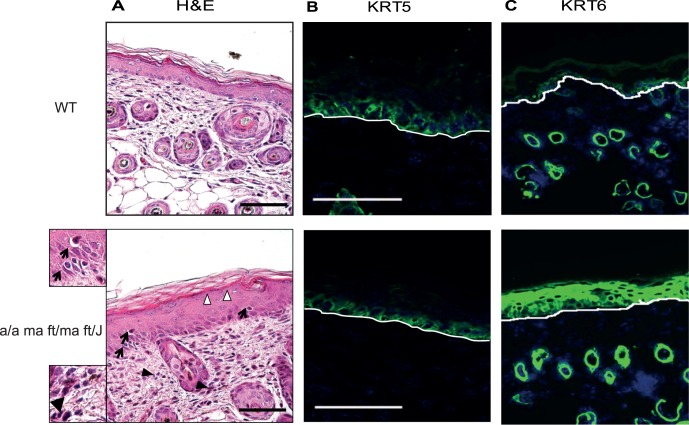
Acanthosis and inflammation in 5-days-old *a/a ma ft/ma ft/J* skin. **A.** Dorsal skin of P5 C57blk6/J wild type (WT) and flaky tail (*a/a ma ft/ma ft/J*) mice was collected, fixed in PFA 4% and embedded in paraffin. H&E staining was performed and sections were visualized with a 20x lens. Thickening of the epidermis, hypogranulosis (white arrowheads), lymphocytic exocytosis (arrows) and inflammatory infiltrates (black arrowheads) are observed. Magnification of two representative fields is presented in order to distinguish inflammatory infiltrates in the *a/a ma ft/ma ft/J* mouse skin. (**B** and **C**)**.** Dorsal skin biopsies were embedded in OCT and cryosections were prepared. Immunofluorescence was performed using antibodies against keratin 5 (KRT5) **(B)** and keratin 6 (KRT6) **(C)**. Fluorescence was visualized with a 20x lens (LSM 700 Zeiss confocal microscope (B) or Zeiss Axiovision microscope (C)). Scale bars = 50 µm.

This study was performed on five days old pups because this is the earliest time point when the “flaky tail” phenotype is present and well manifest [1]. Histological analysis of flaky tail (*a/a ma ft/ma ft/J*) dorsal skin showed thickening of the epidermis with acanthosis, hypogranulosis, and mild hyperkeratosis. Lymphocytic exocytosis and mild spongiosis were also observed (Fig. 1A). Immunohistochemistry experiments showed no difference for keratin 5 expression between *a/a ma ft/ma ft/J* and WT pups, whereas increased keratin 6 staining of the *a/a ma ft/ma ft/J* epidermis demonstrated an abnormal differentiation state and indicated hyperproliferation (Fig. 1B and C). Together, these findings indicate a reactive inflammatory epidermitis in *a/a ma ft/ma ft/J* mice. Therefore, inflammatory pathways were analyzed.

### Increased Levels of Proinflammatory Cytokines and Langerin+ cell Population in Flaky Tail Mouse Skin

**Figure 2:**
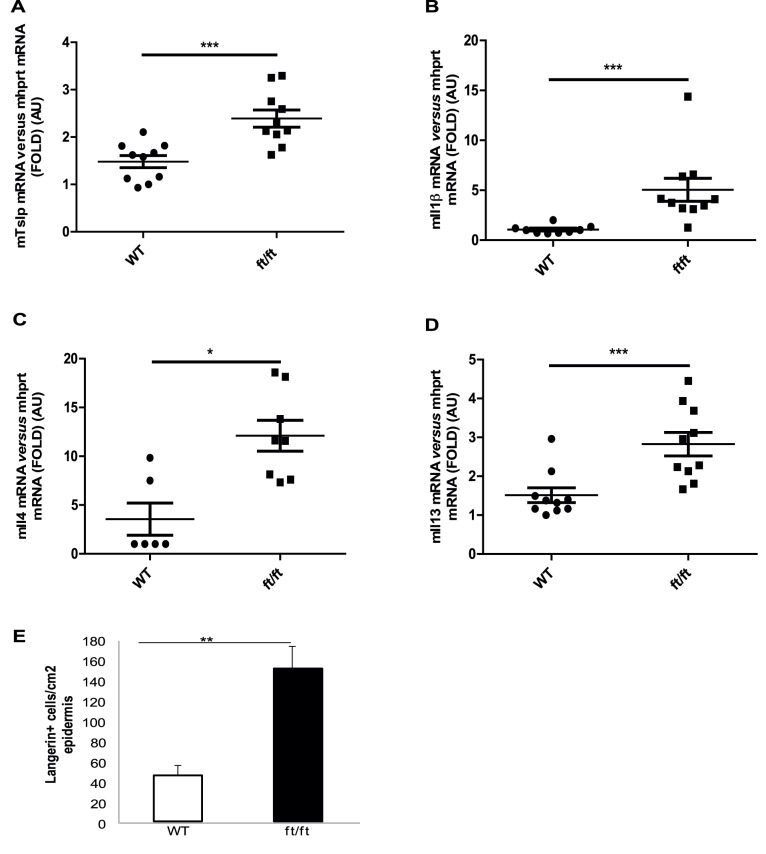
Analysis of proinflammatory and Th2-derived cytokines in *a/a ma ft/ma ft/J* mouse skin. Total RNAs were extracted from dorsal skin of P5 WT and *a/a ma ft/ma ft/J (ft/ft)* mice, and cDNAs were generated. Real-time PCRs were performed using primers specific for (**A**) *Tslp*, (**B**) *Il1β*, (**C**) *Il4* and (**D**) *Il13*. Ct values were normalized with the hprt house-keeping gene. The experiment was realized in groups of 10 mice. (E) Langerin positive cells stained in WT and *a/a ma ft/ma ft/J (ft/ft)* mice skin biopsies were quantified. Statistically significant differences were calculated with the student T-test (p<0.05 * and p<0.001 ***).

Exposure of the epidermis to environmental stimuli, such as allergens or microbial agents, and alteration of the barrier function, lead to activation of resident cells, i.e. keratinocytes, mastocytes and dendritic cells, which initiate an inflammatory cascade. Early inflammatory responses include secretion of IL1β, TNFα and TSLP [23], [24], [25], [26], which were measured in *a/a ma ft/ma ft/J* skin. mRNAs were analyzed by real time PCR and normalized to two different housekeeping genes, *hprt* and *gadph*. The data were similar (data not shown), therefore we present here only the normalization to *hprt*. *Il1β* and *Tslp* mRNA levels were significantly increased in *a/a ma ft/ma ft/J* mouse dorsal skin, whereas *Tnfα* mRNA levels remained unchanged when compared to WT mice (Fig. 2A and B and not shown). Further, main Th2-derived cytokines were measured in order to investigate early polarization, generally consequently to TSLP upregulation [26]. Indeed, *Il4* and *Il13* mRNAs were significantly increased in the *a/a ma ft/ma ft/J* mice, although the variability between individuals was noteworthy (Fig. 2C and D). The mRNA levels of Th1 or Th17-derived cytokines, such as *Ifnγ* and *Il17*a respectively, were unchanged at 5-days old *a/a ma ft/ma ft/J* mice compared to WT pups (data not shown).

Th2-polarization, and consequently of AD, is associated with LCs in the site of lesion. Our immunohistochemistry findings show that *a/a ma ft/ma ft/J* skin hosts significantly higher population of langerin positive cells, compared to WT mice (Fig. 2E and data not shown).These data suggest that barrier disruption in the *a/a ma ft/ma ft/J* mouse leads to a Th2-polarization and to increased number LCs, reminiscent of AD.

### Activation of NFκB Signaling in the Flaky Tail Mice

**Figure 3:**
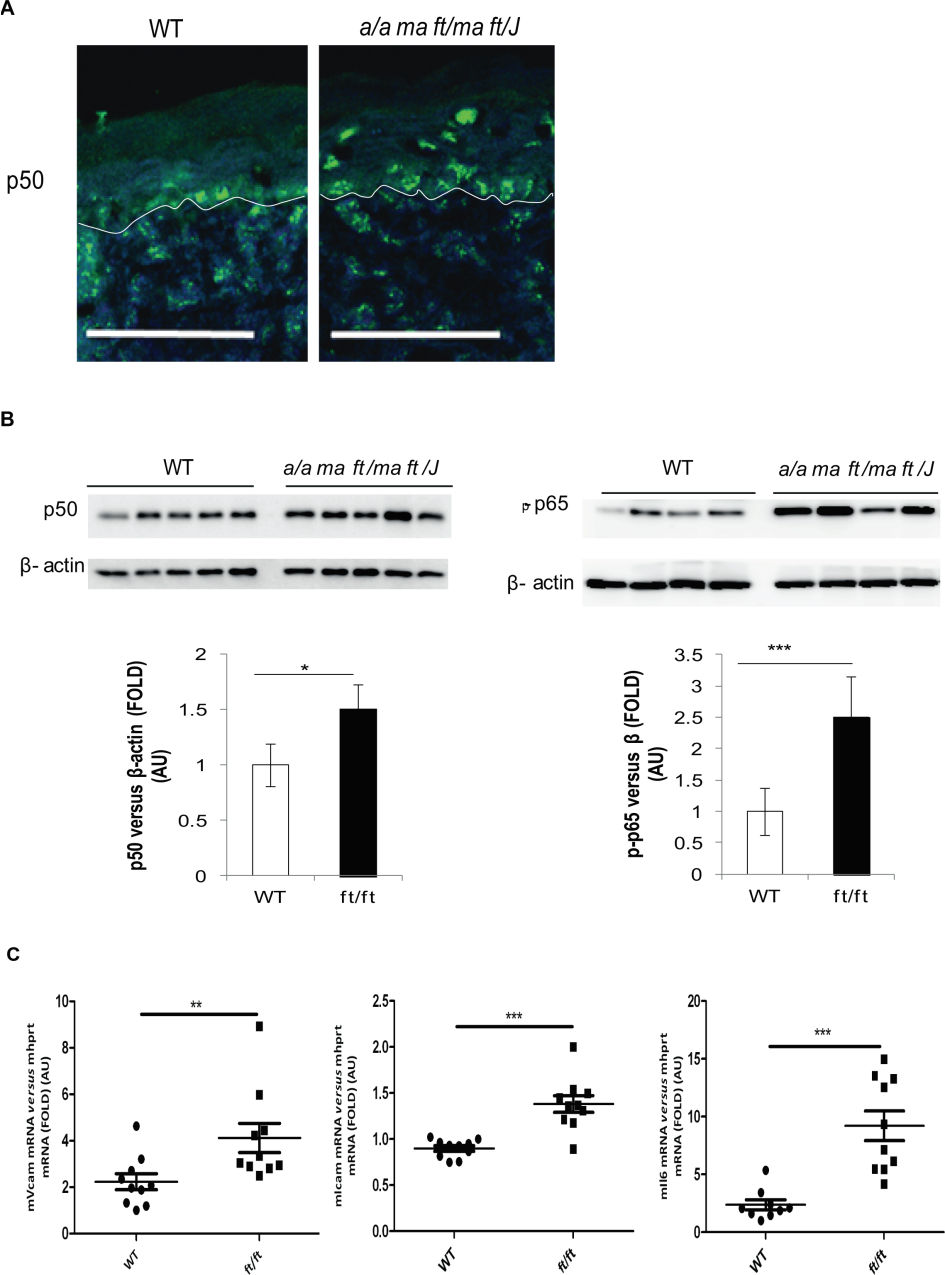
NFκB signaling in *a/a ma ft/ma ft/J* mice. **A.** P5 WT and *a/a ma ft/ma ft/J* mouse skin cryosections were immunostained with an anti-p50 antibody. Fluorescence was visualized with a 20x lens. Scale bar = 50 µm. (**B**). Epidermal protein extracts from P5 WT and *a/a ma ft/ma ft/J (ft/ft)*mice were submitted to WB, performed with anti-p50, anti-p-p65 and anti-β-actin antibodies, and detected by chemiluminescence. Quantification of p50 and p-p65 expression normalized to β-actin is represented in histograms. (**C**). Total RNAs were extracted from WT and *a/a ma ft/ma ft/J (ft/ft)* mice dorsal skin, and real-time PCRs were performed using primers specific for *Vcam*, *Icam*, and *Il6*. Ct values were normalized to *hprt*. The experiment was realized in groups of 10 mice and statistically significant differences were calculated with the student T-test (**p<0.1 and ***p<0.001).

IL1β activates MAPK and NFκB pathways. TSLP is upstream of Stat5, although it can also signal *via* NFκB, MAPK and Stat3 pathways, according to the cell type and the biological system settings [27], [28]. We investigated NFκB activation, because this is a pathway typically activated during AD onset [29]. NFκB p50 subunit had a pronounced nuclear localization in *a/a ma ft/ma ft/J* basal keratinocytes compared to WT mice, and it was present suprabasally in contrast to WT samples (Fig. 3A). Western Blotting (WB) experiments confirmed increase of p50 and, to a greater extent, phospho-p65 expression in *a/a ma ft/ma ft/J* epidermis (Fig. 3B). Further, known NFκB target gene expression was increased in the *a/a ma ft/ma ft/J* mice, such as *Vcam*, *Icam* and *Il6* mRNAs (Fig. 3C). VCAM and ICAM are upregulated after epidermal inflammation and during AD to favor immune cell migration into the affected area [16]. These results indicate that AD-like symptoms in *a/a ma ft/ma ft/J* mice may result from early NFκB activation.

### Increased SPRR2 Expression in the *a/a ma ft/ma ft/J* Mice

**Figure 4:**
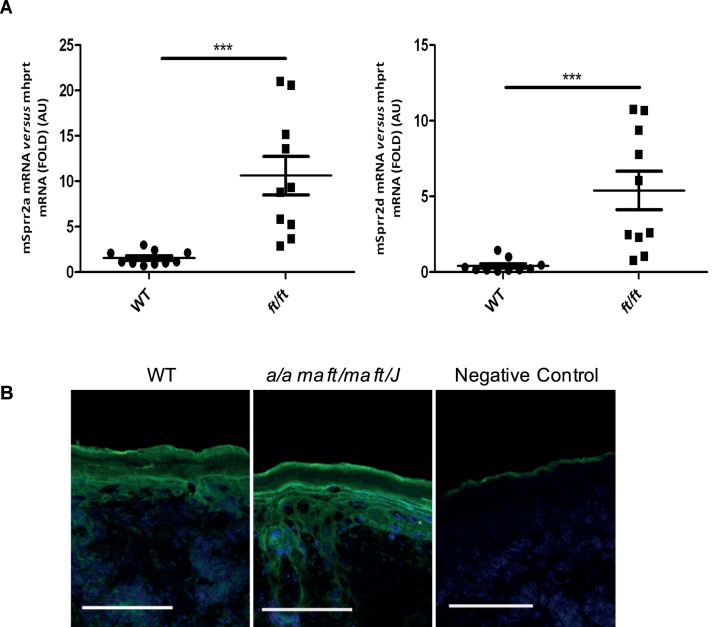
Increased Sprr2 expression in *a/a ma ft/ma ft/J* mouse epidermis. A. cDNA was prepared from P5 WT and *a/a ma ft/ma ft/J (ft/ft)* mice, real-time PCRs were performed using primers specific for *Sprr2a* and *Sprr2d*. Ct values were normalized with the *hprt* house-keeping gene. The experiment was realized in groups of 10 mice and statistically significant differences were calculated with the student T-test (***p<0.001). B. P5 WT and *a/a ma ft/ma ft/J* mouse skin cryosections were immunostained with an anti-SPRR2 antibody. Fluorescence was visualized with a 20x lens. Scale bar = 50 µm.

Il6 upregulation and barrier disruption in *a/a ma ft/ma ft/J* mice is reminiscent of injury-induced stress conditions which lead to changes in tissue homeostasis. Sprrs are stress-induced epidermal differentiation components and are upregulated under different pathological conditions [17], [30], [31], [32]. *Sprr* mRNA expression analysis revealed a strong upregulation of *Sprr2a* and *Sprr2d* genes in the *a/a ma ft/ma ft/J* mice (Fig. 4A). Immunohistochemistry of Sprr2 proteins was suggestive of increased expression in *a/a ma ft/ma ft/J* suprabasal keratinocytes (Fig. 4B). In contrast, the expression of epidermal differentiation proteins involucrin, loricrin, repetin and cornulin remained unchanged (data not shown). Sprr2-specific upregulation in *a/a ma ft/ma ft/J* suggests an epidermal stress response to injury.

### Stat Pathway is Activated in the Flaky Tail Mice Skin

**Figure 5:**
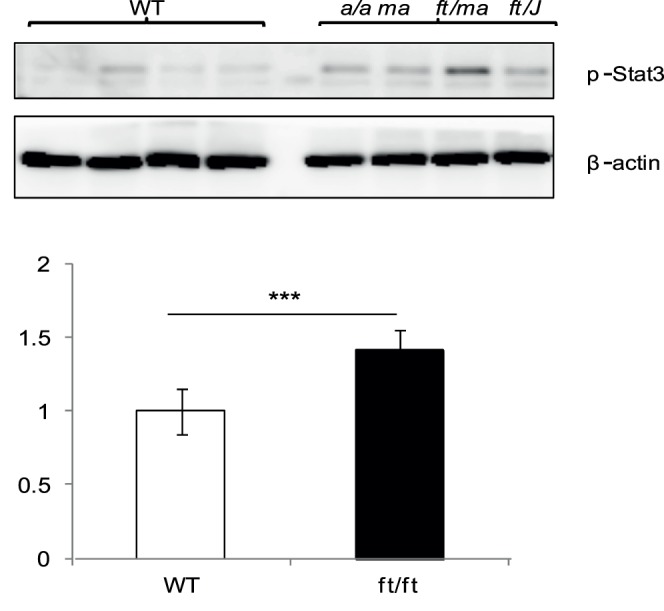
Stat3 phosphorylation in *a/a ma ft/ma ft/J* mouse epidermis. Protein skin extracts from P5 WT and *a/a ma ft/ma ft/J (ft/ft)* mice were submitted to WB, performed with rabbit anti-phospho-Stat3 and anti-actin antibodies, and detected by chemiluminescence. Quantification of p-Stat3 expression normalized to β-actin is represented in histograms. Statistical differences were calculated with the student T-test (***p<0.001).

Both Nrf2 and Stat3 pathways are known to transcriptionally regulate Sprr2 expression [17], [33], [34]. As indicated above, Il6, which signals through Stat3 pathway, was elevated in *a/a ma ft/ma ft/J* epidermis. Thus, we tested whether Sprr2 upregulation was dependent on Stat3 phosphorylation. Stat3 was significantly phosphorylated in *a/a ma ft/ma ft/J* mouse epidermal protein extracts (Fig. 5), suggesting that Sprr2 increase in the *a/a ma ft/ma ft/J* mouse is a consequence of Stat3 activation. Analysis of the Nrf2 pathway by measuring known target genes Nqo1 (NAD(P)H dehydrogenase quinone 1) and Gsta3 (glutathione S-transferase A3) [35] did not reveal changes (data not shown), indicating that Nrf2-dependent Reactive Oxygen Species (ROS) signaling is not activated in 5-days old *a/a ma ft/ma ft/J* mice.

### Increased SPRR2 Expression in the K14-TSLP tg Mice

**Figure 6:**
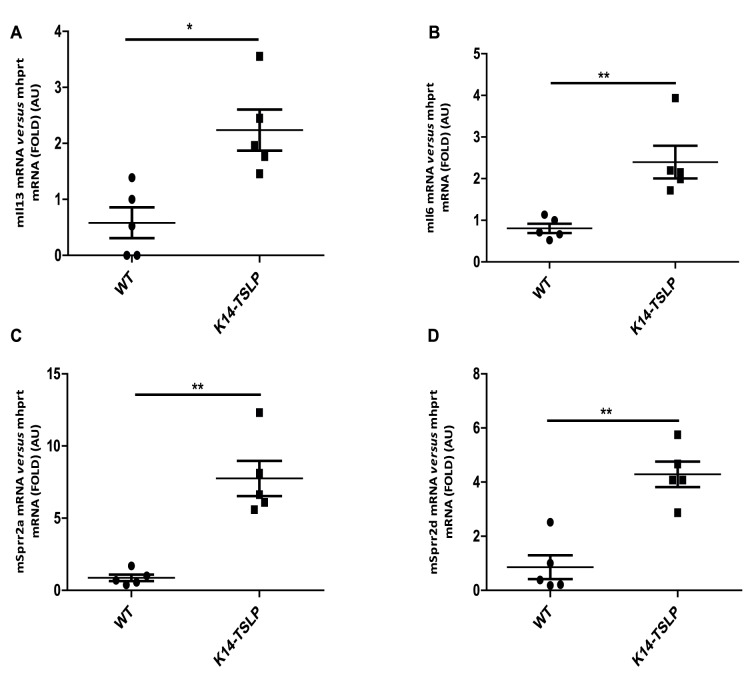
Real time PCR analysis of gene expression in K14-TSLP tg mouse skin. cDNA was prepared from P5 WT and *K14-TSLP tg* mice, and real-time PCRs were performed using primers specific for (A) *Il13*, (B) *Il6*, (C) *Sprr2a* and (D) *Sprr2d*. The experiment was realized in groups of 5 WT and 5 *K14-TSLP* P5 mice and statistically significant differences were calculated with the student T-test (*p<0.05 and **p<0.01).

Sprr2 transcription can be also regulated by IL4/IL13/Stat6, as described in an experimental asthma mouse model [36]. TSLP being a main inducer of Th2-polarization, we questioned whether Sprr2 are downstream of TSLP and/or Th2-cytokines. Therefore, skin biopsies were obtained from P5 transgenic mice specifically overexpressing TSLP in the epidermis (K14-TSLP tg), which develop a spontaneous AD [37], [38]. As expected, *Il4* and *Il13* mRNAs were increased, as well as *Il6*, *Sprr2a* and *Sprr2d* mRNAs (Fig. 6A to D and data not shown). Filaggrin mRNA levels were not modified, showing that IL4, IL13 and Sprr2 regulation is independent on filaggrin expression, and downstream of TSLP (data not shown).

### Corneodesmosomal Desquamation in the Flaky Tail Mice Occurs Normally

Human IV is characterized by a retention hyperkeratosis without hyperproliferation of keratinocytes [33]. Consequently, we tested whether the presence of scales on flaky tail skin is a consequence of abnormal desquamation; major corneodesmosomal components and their proteolytic enzymes were analyzed. mRNA and protein evaluation showed no significant differences of corneodesmosin, desmoglein and desmocollin synthesis between *a/a ma ft/ma ft/J* and WT mice. Further, Spink5 expression was not altered and the proteolytic enzymes, kallikrein 5 and kallikrein 7 were equally expressed and active in both mouse types (Fig. S1 and data not shown). Our results are in line with recent data reporting that KLK5 activity and KLK7 expression are increased only in adult, and not in newborn *a/a ma ft/ma ft/J* mice, when the pH is elevated, probably due to low histidine levels (Table S6 in File S1). Combination of these data suggest that abnormal expression of SC proteases is a secondary consequence of inflammation and altered pH levels, rather than a characteristic directly resulting from the mouse phenotype [39].

### IL1β and SPRR2a mRNA Levels are Increased in AD, but not in IV Patients

**Figure 7:**
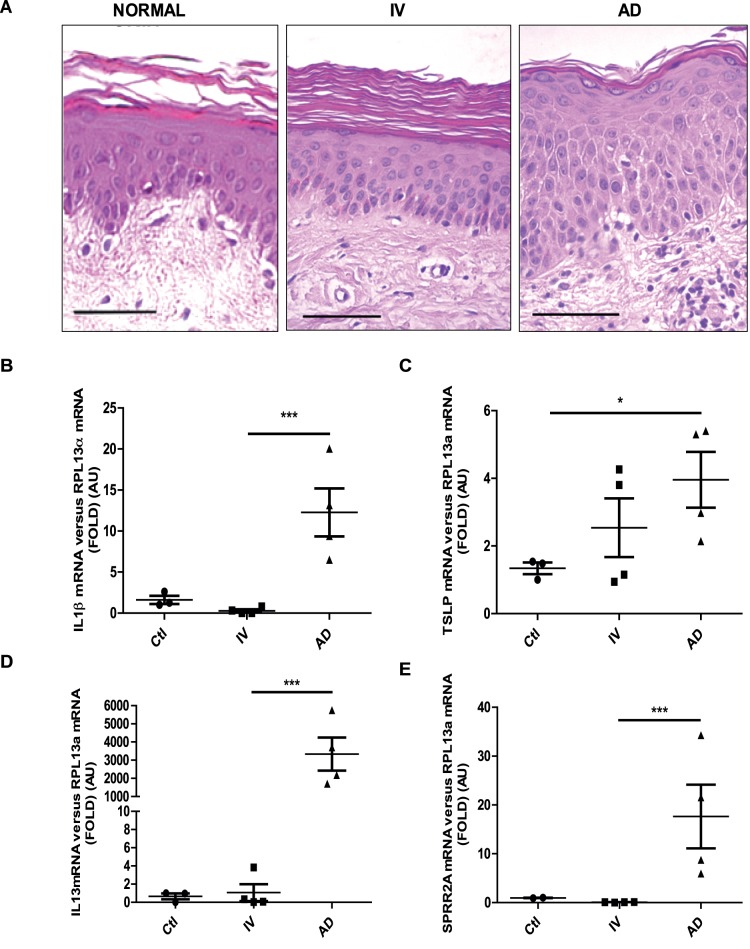
Comparison of healthy, IV and AD skin. **A.** Skin biopsies from healthy, IV and AD-patients were collected, fixed in PFA 4% and embedded in paraffin. H&E staining was performed and sections were visualized with a 20x lens. Scale bar = 50 µm. **B–E.** cDNA were prepared from healthy, IV and AD-patient skin biopsies and real-time PCRs were performed using primers specific for (B) *IL1β*, (C) *TSLP* (D) *IL13* and (E) *SPRR2a*. The experiment was realized in groups of 4 patients and statistically significant differences were calculated with the student T-test (*p<0.05 and ***p<0.001).

To correlate our findings with the human pathology, skin biopsies form AD and IV patients were analyzed. Human AD epidermis shows acanthosis, exocytosis, spongiosis and hyperproliferation. In contrast, IV epidermis is not inflamed, the granular layer is attenuated or absent and SC is orthohyperkeratotic (Fig. 7A and Table S1 in File S1). In this line, RT-PCR analysis showed increased *IL1β* mRNA levels only in AD patients, and elevated *TSLP* mRNA levels in both AD and IV patients, although marked discrepancies between individuals were observed (Fig. 7B and C). Further, as expected, *IL13* mRNA was increased only in AD patients. Finally, AD, but not IV, is associated with upregulated *SPRR2a* transcription (Fig. 7D and E). These data show that the *a/a ma ft/ma ft/J* mouse phenotype reproduces several biological aspects of human AD.

## Discussion

Here we present the first thorough analysis of inflammatory pathways in newborn *a/a ma ft/ma ft/J* mice and demonstrate that they adopt very early an atopic phenotype, well correlating with human atopic dermatitis (Table S5 in File S1).

Increased levels of both IL1β and TSLP are expressed early after birth without any mechanical injury or allergen challenge. Our results on *Il1β* mRNA levels corroborate, confirm and extend very recent data reporting increased IL1 levels in AD patients carrying FLG mutations [40]. The *a/a ma ft/ma ft/J* mice lack filaggrin, and bear a 215kDa unprocessed peptide likely devoid of a dominant negative effect as heterozygous animals appear normal. Except from the FLG loss-of-function mutation, *a/a ma ft/ma ft/J* mice carry the matted coat mutation (*ma/ma*), which is responsible for abnormal coat and not yet characterized [1], [41]. Although the exact role of the *ma/ma* in the AD-like phenotype is not elucidated, a few studies having used a flaky tail mouse devoid of the matted mutation (*flg^ft^/flg^ft^/J*), are informative: 8 week old *flg^ft^/flg^ft^/J* mice have normal TEWL, but present mild orthokeratotic hyperkeratosis and acanthosis, lymphocytic infiltrates in the skin and high Il1β and IlRA mRNA levels (Fallon et al. 2009; Kezic et al. 2012). Interestingly, in *a/a ma ft/ma ft/J* embryos (E17.5) the barrier function is already defective and there is peridermal retention [8]. These observations suggest that filaggrin deficiency alone is able to induce mild inflammation [2] and predispose to allergic response [2], [42]. On the other hand, the *matted coat* mutation is likely to amplify the dysfunction of the barrier, and to accelerate/enhance the inflammatory reaction, eventually after changes in upper epidermis biophysical properties [5], [8], [18], [39](Table S6 in File S1). Beyond filaggrin deficiency, mechanical or genetic barrier disruption over time can lead to increased expression of proinflammatory cytokines and recruitment of LCs on the site of lesion [22], [23], [24], [25], [43], according to environmental and experimental conditions [5], [18].

Our data reveal that the *a/a ma ft/ma ft/J* mouse model, instead of replicating IV features only, presents also acanthosis and inflammation [33]. Indeed, at birth, humans with semi-dominant IV have normal skin that becomes dry and rough after 3–6 months [44], [45]. On the other hand, 45% of AD patients develop symptoms within the first 6 months of life [15], [46]. Resemblance between the flaky tail mouse and human AD is further confirmed by Th2-derived cytokine expression in *a/a ma ft/ma ft/J* skin, which provides evidence of an early spontaneous engagement into a hypersensitivity reaction.

IL1β signals through NFκB and MAPK pathways, and promotes inflammation through TSLP and IL6, and cell migration through regulation of cell adhesion molecules, and various chemokines [47], [48]. Accordingly, in *a/a ma ft/ma ft/J* epidermis, the NFκB pathway is activated as demonstrated by accumulation of p50 and phospho-p65 in epidermis, and by increased expression of VCAM, ICAM and IL6. The NFκB pathway in AD mediates proinflammatory cytokine signals, and TLRs are activated subsequent to microbial colonization [29], [49].

TSLP is an epithelial-derived cytokine involved in immune responses, through naïve T-cells homeostasis regulation, cell differentiation and migration [50], [51], [52]. It is tightly associated with inflammation and hypersensitivity responses because it triggers B-cells differentiation and Th2-polarization [53], [54], [55]. Accordingly, *Il4* and *Il13* mRNAs are increased in *a/a ma ft/ma ft/J* skin revealing another typical early sign of atopy. TSLP signals through its TSLPR heterodimeric receptor - absent from keratinocytes - and STAT5 [56].

TSLP induces IL6 production from human airway smooth muscle cells through MAPK/Stat3 *in vitro* [28]. Our data, combining *a/a ma ft/ma ft/J* and K14-TSLP skin analysis, suggest that increased *Il6* mRNA levels in *a/a ma ft/ma ft/J* may be a consequence of IL1β and TSLP signaling. IL6 in skin is secreted by keratinocytes, fibroblasts and T-cells, especially the Th17-subset that induces inflammation and defense against pathogens [57]. Increased IL6 production from T-cells has been found in AD patients [58], which may be associated with its property to promote Th2-cell differentiation [59]. It is noteworthy that IL6, according to the cytokine milieu, can favor Th17 development [60]. Along these lines, Th17-subset and their effector cytokines are increased in adult *a/a ma ft/ma ft/J* mice [18]. Presence of Th17-regulators in newborn *a/a ma ft/ma ft/J* skin suggests 1. a modulation of the cytokine pool with age, and 2. that qualitative and quantitative modifications in the cytokine milieu over time and environmental conditions might be responsible for the changing phenotypical characteristics of *a/a ma ft/ma ft/J* under various experimental conditions. We propose that early IL6 increase also promotes morphological changes in *a/a ma ft/ma ft/J* epidermis, and induces SPRR2A expression.

SPRR proteins, are encoded by genes located on EDC, and individual members are upregulated in different tissues under stress conditions or injury [17], [30], [61], [62], [63], [64]. In epidermis, *Sprr2A* and *Rptn* gene transcription is upregulated in Klf4-null mice, characterized by impaired barrier function, without preventing lethality [32]. Further, *Sprr2D*, *Sprr2H* and *Rptn* transcripts are increased in loricrin-deficient mice, characterized by a transient erythroderma [65]. Non-coordinate regulation of *Sprr1/2* genes in various tissues after injury suggests that *Sprr1/2* subfamily genes play specific roles linked with tissue-remodeling and cell survival. Besides their biomechanical function, SPRRs detoxify ROS in various epithelia *via* their Cys residues [66], [67].

Sprr2 are positively regulated through IL6/Stat3 and IL6/MAPK signaling in cardiomyocytes, after ischemic injury, and in biliary epithelial cells after bile duct ligation [17], [31]. In *a/a ma ft/ma ft/J*, Sprr2 are a potential IL6/Stat3 target and they may compensate for filaggrin loss, or function as initiators of an attempt to repair. We propose that Sprr2 gene regulation in *a/a ma ft/ma ft/J* mice is downstream of pathways activated by TSLP/IL6/IL4/IL13 ligands.

In sum, our study reveals that filaggrin deficiency in the appropriate genetic background leads to spontaneous dermatitis associated with increased IL1β, Th2-related cytokines and activated NFκB signalling. This report is complementary to previous studies, because it presents novel findings and provides correlations with biological features of human AD, therefore validating the flaky tail mouse as an interesting early AD model. Indirectly, our results suggest that very early therapeutic intervention in atopic dermatitis associated with filaggrin deficiency may be essential for disease prevention. Along these lines, we are currently analysing a large cohort of human newborns during the first 2 years of life in order to correlate genetic predisposition and cytokine profiles (S. Christen and D. Hohl, unpublished observations).

## Supporting Information

Figure S1 **Corneodesmosomal components expression in unchanged between WT and** ***a/a ma ft/ma ft/J*** **mice. (A).** P5 WT and *a/a ma ft/ma ft/J* mouse skin cryosections were immunostained with antibodies anti-corneodesmosin (Csdn), anti-desmoglein (Dsg) and anti-desmocollin (Dsc). Fluorescence was visualized with a 20x lens. **(B).** Epidermal insoluble and soluble protein extracts from P5 WT and *a/a ma ft/ma ft/J* mice were submitted to WB, performed with anti-corneodesmosin (Csdn), anti-desmocollin (Dsc), anti-desmoglein (Dsg), and anti-β-actin antibodies, and detected by chemiluminescence. Ponceau S staining shows equal loading for the insoluble protein fraction.
(EPS)

File S1 **Supporting Tables and References.**
(DOCX)

## References

[1] Presland RB, Boggess D, Lewis SP, Hull C, Fleckman P, et al (2000) Loss of normal profilaggrin and filaggrin in flaky tail (ft/ft) mice: an animal model for the filaggrin-deficient skin disease ichthyosis vulgaris. J Invest Dermatol 115: 1072–1081. 11121144 10.1046/j.1523-1747.2000.00178.x

[2] Fallon PG, Sasaki T, Sandilands A, Campbell LE, Saunders SP, et al (2009) A homozygous frameshift mutation in the mouse Flg gene facilitates enhanced percutaneous allergen priming. Nat Genet 41: 602–608. 19349982 10.1038/ng.358 PMC2872154

[3] Vercelli D (2009) Of flaky tails and itchy skin. Nat Genet 41: 512–513. 19399034 10.1038/ng0509-512

[4] Smith FJ, Irvine AD, Terron-Kwiatkowski A, Sandilands A, Campbell LE, et al (2006) Loss-of-function mutations in the gene encoding filaggrin cause ichthyosis vulgaris. Nat Genet 38: 337–342. 16444271 10.1038/ng1743

[5] Moniaga CS, Egawa G, Kawasaki H, Hara-Chikuma M, Honda T, et al (2010) Flaky tail mouse denotes human atopic dermatitis in the steady state and by topical application with Dermatophagoides pteronyssinus extract. Am J Pathol 176: 2385–2393. 20304960 10.2353/ajpath.2010.090957 PMC2861103

[6] Scharschmidt TC, Man MQ, Hatano Y, Crumrine D, Gunathilake R, et al.. (2009) Filaggrin deficiency confers a paracellular barrier abnormality that reduces inflammatory thresholds to irritants and haptens. J Allergy Clin Immunol 124: 496–506, 506 e491–496. 10.1016/j.jaci.2009.06.046 PMC2881668 19733297

[7] Kawasaki H, Nagao K, Kubo A, Hata T, Shimizu A, et al.. (2012) Altered stratum corneum barrier and enhanced percutaneous immune responses in filaggrin-null mice. J Allergy Clin Immunol 129: 1538–1546 e1536. 10.1016/j.jaci.2012.01.068 22409988

[8] Okano J, Lichti U, Mamiya S, Aronova M, Zhang G, et al (2012) Increased retinoic acid levels through ablation of Cyp26b1 determine the processes of embryonic skin barrier formation and peridermal development. J Cell Sci 125: 1827–1836. 22366455 10.1242/jcs.101550 PMC3346831

[9] Dale BA, Presland RB, Lewis SP, Underwood RA, Fleckman P (1997) Transient expression of epidermal filaggrin in cultured cells causes collapse of intermediate filament networks with alteration of cell shape and nuclear integrity. J Invest Dermatol 108: 179–187. 9008231 10.1111/1523-1747.ep12334205

[10] Sandilands A, Smith FJ, Irvine AD, McLean WH (2007) Filaggrin's fuller figure: a glimpse into the genetic architecture of atopic dermatitis. J Invest Dermatol 127: 1282–1284. 17502856 10.1038/sj.jid.5700876

[11] Candi E, Schmidt R, Melino G (2005) The cornified envelope: a model of cell death in the skin. Nat Rev Mol Cell Biol 6: 328–340. 15803139 10.1038/nrm1619

[12] Mildner M, Jin J, Eckhart L, Kezic S, Gruber F, et al (2010) Knockdown of filaggrin impairs diffusion barrier function and increases UV sensitivity in a human skin model. J Invest Dermatol 130: 2286–2294. 20445547 10.1038/jid.2010.115

[13] O'Regan GM, Sandilands A, McLean WH, Irvine AD (2008) Filaggrin in atopic dermatitis. J Allergy Clin Immunol 122: 689–693. 18774165 10.1016/j.jaci.2008.08.002

[14] Bieber T (2008) Atopic dermatitis. N Engl J Med 358: 1483–1494. 18385500 10.1056/NEJMra074081

[15] Bieber T (2010) Atopic dermatitis. Ann Dermatol 22: 125–137. 20548901 10.5021/ad.2010.22.2.125 PMC2883413

[16] Leung DY, Boguniewicz M, Howell MD, Nomura I, Hamid QA (2004) New insights into atopic dermatitis. J Clin Invest 113: 651–657. 14991059 10.1172/JCI21060 PMC351324

[17] Pradervand S, Yasukawa H, Muller OG, Kjekshus H, Nakamura T, et al (2004) Small proline-rich protein 1A is a gp130 pathway- and stress-inducible cardioprotective protein. The EMBO journal 23: 4517–4525. 15510217 10.1038/sj.emboj.7600454 PMC526469

[18] Oyoshi MK, Murphy GF, Geha RS (2009) Filaggrin-deficient mice exhibit TH17-dominated skin inflammation and permissiveness to epicutaneous sensitization with protein antigen. J Allergy Clin Immunol 124: 485–493, 493 e481. 10.1016/j.jaci.2009.05.042 PMC2886150 19665780

[19] Sandilands A, Terron-Kwiatkowski A, Hull PR, O'Regan GM, Clayton TH, et al (2007) Comprehensive analysis of the gene encoding filaggrin uncovers prevalent and rare mutations in ichthyosis vulgaris and atopic eczema. Nat Genet 39: 650–654. 17417636 10.1038/ng2020

[20] Obarzanek-Fojt M, Favre B, Kypriotou M, Ryser S, Huber M, et al (2011) Homeodomain-only protein HOP is a novel modulator of late differentiation in keratinocytes. European journal of cell biology 90: 279–290. 21256618 10.1016/j.ejcb.2010.11.001

[21] Livak KJ, Schmittgen TD (2001) Analysis of relative gene expression data using real-time quantitative PCR and the 2(-Delta Delta C(T)) Method. Methods 25: 402–408. 11846609 10.1006/meth.2001.1262

[22] Descargues P, Deraison C, Bonnart C, Kreft M, Kishibe M, et al (2005) Spink5-deficient mice mimic Netherton syndrome through degradation of desmoglein 1 by epidermal protease hyperactivity. Nat Genet 37: 56–65. 15619623 10.1038/ng1493

[23] Angelova-Fischer I, Fernandez IM, Donnadieu MH, Bulfone-Paus S, Zillikens D, et al (2010) Injury to the stratum corneum induces in vivo expression of human thymic stromal lymphopoietin in the epidermis. J Invest Dermatol 130: 2505–2507. 20555350 10.1038/jid.2010.143

[24] Barker JN, Mitra RS, Griffiths CE, Dixit VM, Nickoloff BJ (1991) Keratinocytes as initiators of inflammation. Lancet 337: 211–214. 1670850 10.1016/0140-6736(91)92168-2

[25] Nickoloff BJ, Naidu Y (1994) Perturbation of epidermal barrier function correlates with initiation of cytokine cascade in human skin. J Am Acad Dermatol 30: 535–546. 7512582 10.1016/s0190-9622(94)70059-1

[26] Oyoshi MK, Larson RP, Ziegler SF, Geha RS (2010) Mechanical injury polarizes skin dendritic cells to elicit a T(H)2 response by inducing cutaneous thymic stromal lymphopoietin expression. J Allergy Clin Immunol 126: 976–984, 984 e971–975. 10.1016/j.jaci.2010.08.041 PMC3085022 21050944

[27] Kamekura R, Kojima T, Takashima A, Koizumi J, Ogasawara N, et al (2010) Thymic stromal lymphopoietin induces tight junction protein claudin-7 via NF-kappaB in dendritic cells. Histochemistry and cell biology 133: 339–348. 20077120 10.1007/s00418-009-0674-1

[28] Shan L, Redhu NS, Saleh A, Halayko AJ, Chakir J, et al (2010) Thymic stromal lymphopoietin receptor-mediated IL-6 and CC/CXC chemokines expression in human airway smooth muscle cells: role of MAPKs (ERK1/2, p38, and JNK) and STAT3 pathways. Journal of immunology 184: 7134–7143. 10.4049/jimmunol.0902515 20483734

[29] Nakamura H, Aoki M, Tamai K, Oishi M, Ogihara T, et al (2002) Prevention and regression of atopic dermatitis by ointment containing NF-kB decoy oligodeoxynucleotides in NC/Nga atopic mouse model. Gene therapy 9: 1221–1229. 12215889 10.1038/sj.gt.3301724

[30] Hooper LV, Wong MH, Thelin A, Hansson L, Falk PG, et al (2001) Molecular analysis of commensal host-microbial relationships in the intestine. Science 291: 881–884. 11157169 10.1126/science.291.5505.881

[31] Nozaki I, Lunz JG, 3rd, Specht S, Stolz DB, Taguchi K, et al (2005) Small proline-rich proteins 2 are noncoordinately upregulated by IL-6/STAT3 signaling after bile duct ligation. Lab Invest 85: 109–123. 15558059 10.1038/labinvest.3700213

[32] Segre JA, Bauer C, Fuchs E (1999) Klf4 is a transcription factor required for establishing the barrier function of the skin. Nat Genet 22: 356–360. 10431239 10.1038/11926

[33] Frost P, Van Scott EJ (1966) Ichthyosiform dermatoses. Classification based on anatomic and biometric observations. Arch Dermatol 94: 113–126. 5911500 10.1001/archderm.94.2.113

[34] Schafer M, Farwanah H, Willrodt AH, Huebner AJ, Sandhoff K, et al (2012) Nrf2 links epidermal barrier function with antioxidant defense. EMBO Mol Med 4: 364–379. 22383093 10.1002/emmm.201200219 PMC3403295

[35] Nguyen T, Nioi P, Pickett CB (2009) The Nrf2-antioxidant response element signaling pathway and its activation by oxidative stress. J Biol Chem 284: 13291–13295. 19182219 10.1074/jbc.R900010200 PMC2679427

[36] Zimmermann N, Doepker MP, Witte DP, Stringer KF, Fulkerson PC, et al (2005) Expression and regulation of small proline-rich protein 2 in allergic inflammation. Am J Respir Cell Mol Biol 32: 428–435. 15731505 10.1165/rcmb.2004-0269OC

[37] Dumortier A, Durham AD, Di Piazza M, Vauclair S, Koch U, et al (2010) Atopic dermatitis-like disease and associated lethal myeloproliferative disorder arise from loss of Notch signaling in the murine skin. PLoS One 5: e9258. 20174635 10.1371/journal.pone.0009258 PMC2823782

[38] Yoo J, Omori M, Gyarmati D, Zhou B, Aye T, et al (2005) Spontaneous atopic dermatitis in mice expressing an inducible thymic stromal lymphopoietin transgene specifically in the skin. J Exp Med 202: 541–549. 16103410 10.1084/jem.20041503 PMC2212851

[39] Moniaga CS, Jeong SK, Egawa G, Nakajima S, Hara-Chikuma M, et al (2013) Protease activity enhances production of thymic stromal lymphopoietin and basophil accumulation in flaky tail mice. Am J Pathol 182: 841–851. 23333753 10.1016/j.ajpath.2012.11.039

[40] Kezic S, O'Regan GM, Lutter R, Jakasa I, Koster ES, et al.. (2012) Filaggrin loss-of-function mutations are associated with enhanced expression of IL-1 cytokines in the stratum corneum of patients with atopic dermatitis and in a murine model of filaggrin deficiency. J Allergy Clin Immunol 129: 1031–1039 e1031. 10.1016/j.jaci.2011.12.989 PMC3627959 22322004

[41] Rice RH, Wong VJ, Pinkerton KE, Sundberg JP (1999) Cross-linked features of mouse pelage hair resistant to detergent extraction. Anat Rec 254: 231–237. 9972808 10.1002/(SICI)1097-0185(19990201)254:2<231::AID-AR9>3.0.CO;2-6

[42] Kawasaki H (2010) Filaggrin knock out mice as a tool for understanding the pathogenesis of atopic dermatitis. International Immunology 22(1) iii63 [Short Communication in14th International Congress of Immunology, Kobe, Japan].

[43] Holzmann S, Tripp CH, Schmuth M, Janke K, Koch F, et al (2004) A model system using tape stripping for characterization of Langerhans cell-precursors in vivo. The Journal of investigative dermatology 122: 1165–1174. 15140219 10.1111/j.0022-202X.2004.22520.x

[44] Oji V, Tadini G, Akiyama M, Blanchet Bardon C, Bodemer C, et al (2010) Revised nomenclature and classification of inherited ichthyoses: results of the First Ichthyosis Consensus Conference in Soreze 2009. J Am Acad Dermatol 63: 607–641. 20643494 10.1016/j.jaad.2009.11.020

[45] Okulicz JF, Schwartz RA (2003) Hereditary and acquired ichthyosis vulgaris. Int J Dermatol 42: 95–98. 12708996 10.1046/j.1365-4362.2003.01308.x

[46] Baron SE, Cohen SN, Archer CB, British Association of D (2012) Royal College of General P (2012) Guidance on the diagnosis and clinical management of atopic eczema. Clin Exp Dermatol 37 Suppl 17–12. 22486763 10.1111/j.1365-2230.2012.04336.x

[47] Lee HC, Ziegler SF (2007) Inducible expression of the proallergic cytokine thymic stromal lymphopoietin in airway epithelial cells is controlled by NFkappaB. Proc Natl Acad Sci U S A 104: 914–919. 17213320 10.1073/pnas.0607305104 PMC1783414

[48] Yano S, Banno T, Walsh R, Blumenberg M (2008) Transcriptional responses of human epidermal keratinocytes to cytokine interleukin-1. J Cell Physiol 214: 1–13. 17941080 10.1002/jcp.21300

[49] Tanaka A, Muto S, Jung K, Itai A, Matsuda H (2007) Topical application with a new NF-kappaB inhibitor improves atopic dermatitis in NC/NgaTnd mice. The Journal of investigative dermatology 127: 855–863. 17068475 10.1038/sj.jid.5700603

[50] Chappaz S, Flueck L, Farr AG, Rolink AG, Finke D (2007) Increased TSLP availability restores T- and B-cell compartments in adult IL-7 deficient mice. Blood 110: 3862–3870. 17702899 10.1182/blood-2007-02-074245

[51] Rochman Y, Spolski R, Leonard WJ (2009) New insights into the regulation of T cells by gamma(c) family cytokines. Nat Rev Immunol 9: 480–490. 19543225 10.1038/nri2580 PMC2814538

[52] Fernandez MI, Heuze ML, Martinez-Cingolani C, Volpe E, Donnadieu MH, et al (2011) The human cytokine TSLP triggers a cell-autonomous dendritic cell migration in confined environments. Blood 118: 3862–3869. 21772055 10.1182/blood-2010-12-323089

[53] Soumelis V, Reche PA, Kanzler H, Yuan W, Edward G, et al (2002) Human epithelial cells trigger dendritic cell mediated allergic inflammation by producing TSLP. Nat Immunol 3: 673–680. 12055625 10.1038/ni805

[54] Demehri S, Turkoz A, Manivasagam S, Yockey LJ, Turkoz M, et al (2012) Elevated epidermal thymic stromal lymphopoietin levels establish an antitumor environment in the skin. Cancer Cell 22: 494–505. 23079659 10.1016/j.ccr.2012.08.017 PMC3480666

[55] Di Piazza M, Nowell CS, Koch U, Durham AD, Radtke F (2012) Loss of Cutaneous TSLP-Dependent Immune Responses Skews the Balance of Inflammation from Tumor Protective to Tumor Promoting. Cancer Cell 22: 479–493. 23079658 10.1016/j.ccr.2012.08.016

[56] Quentmeier H, Drexler HG, Fleckenstein D, Zaborski M, Armstrong A, et al (2001) Cloning of human thymic stromal lymphopoietin (TSLP) and signaling mechanisms leading to proliferation. Leukemia 15: 1286–1292. 11480573 10.1038/sj.leu.2402175

[57] Jutel M, Akdis CA (2011) T-cell subset regulation in atopy. Curr Allergy Asthma Rep 11: 139–145. 21271314 10.1007/s11882-011-0178-7 PMC3047206

[58] Toshitani A, Ansel JC, Chan SC, Li SH, Hanifin JM (1993) Increased interleukin 6 production by T cells derived from patients with atopic dermatitis. The Journal of investigative dermatology 100: 299–304. 8440909 10.1111/1523-1747.ep12469875

[59] Diehl S, Rincon M (2002) The two faces of IL-6 on Th1/Th2 differentiation. Mol Immunol 39: 531–536. 12431386 10.1016/s0161-5890(02)00210-9

[60] Tesmer LA, Lundy SK, Sarkar S, Fox DA (2008) Th17 cells in human disease. Immunol Rev 223: 87–113. 18613831 10.1111/j.1600-065X.2008.00628.x PMC3299089

[61] Bonilla IE, Tanabe K, Strittmatter SM (2002) Small proline-rich repeat protein 1A is expressed by axotomized neurons and promotes axonal outgrowth. J Neurosci 22: 1303–1315. 11850458 10.1523/JNEUROSCI.22-04-01303.2002 PMC6757578

[62] Greco MA, Lorand L, Lane WS, Baden HP, Parameswaran KN, et al (1995) The pancornulins: a group of small proline rich-related cornified envelope precursors with bifunctional capabilities in isopeptide bond formation. The Journal of investigative dermatology 104: 204–210. 7829876 10.1111/1523-1747.ep12612759

[63] Mueller A, O'Rourke J, Grimm J, Guillemin K, Dixon MF, et al (2003) Distinct gene expression profiles characterize the histopathological stages of disease in Helicobacter-induced mucosa-associated lymphoid tissue lymphoma. Proceedings of the National Academy of Sciences of the United States of America 100: 1292–1297. 12552104 10.1073/pnas.242741699 PMC298766

[64] Stern LE, Erwin CR, Falcone RA, Huang FS, Kemp CJ, et al (2001) cDNA microarray analysis of adapting bowel after intestinal resection. J Pediatr Surg 36: 190–195. 11150463 10.1053/jpsu.2001.20050

[65] Koch PJ, de Viragh PA, Scharer E, Bundman D, Longley MA, et al (2000) Lessons from loricrin-deficient mice: compensatory mechanisms maintaining skin barrier function in the absence of a major cornified envelope protein. J Cell Biol 151: 389–400. 11038185 10.1083/jcb.151.2.389 PMC2192642

[66] Vermeij WP, Alia A, Backendorf C (2011) ROS quenching potential of the epidermal cornified cell envelope. The Journal of investigative dermatology 131: 1435–1441. 21248766 10.1038/jid.2010.433

[67] Vermeij WP, Backendorf C (2010) Skin cornification proteins provide global link between ROS detoxification and cell migration during wound healing. PLoS One 5: e11957. 20689819 10.1371/journal.pone.0011957 PMC2914756

