# Supplementary material for: Spontaneous Atopic Dermatitis-Like Symptoms in a/a ma ft/ma ft/J Flaky Tail Mice Appear Early after Birth
Source: PLoS One. 2013 Jul 3;8(7):e67869. doi: 10.1371/journal.pone.0067869 (PMC3700905; doi:10.1371/journal.pone.0067869)
Supplement: File S1 — Supporting Tables and References. (DOCX) [file pone.0067869.s002.docx]

**SUPPORTING INFORMATION**

**Table S1:** Patient clinical information

| **Patient** | **Sex, date of birth** | **Biopsy location** | **Keratohyalin granules** | **Disease severity** | **FLG mutations** |
| --- | --- | --- | --- | --- | --- |
| **IV1** | F, 1968 | leg | thin SG | medium | **R501X** WT  **2282del4** WT  **R2447X** WT |
| **IV2** | F, 1976 | leg | thin SG | medium | **R501X** N/A  **2282del4** N/A  **R2447X** N/A |
| **IV3** | M, 1974 | leg | absent SG | severe | **R501X** HET  **2282del4** WT  **R2447X** HET |
| **IV4** | F, 1932 | leg | absent SG | severe | **R501X**  HET  **2282del4** HET  **R2447X** WT |
| **AD1** | F, 1980 | leg | thin SG | severe | **R501X**  WT  **2282del4** WT  **R2447X** WT |
| **AD2** | F, 1973 | arm | thin SG | severe | **R501X** HET  **2282del4** WT  **R2447X** WT |
| **AD3** | M, 1957 | arm | normal SG | severe | **R501X**  WT  **2282del4** WT  **R2447X** WT |
| **AD4** | F, 1953 | abdomen | thin SG | medium-severe | **R501X**  HET  **2282del4** WT  **R2447X** WT |

**Table S2:** Real time PCR mouse primers used in this study

| **Target Gene** | **Forward** | **Reverse** | **Gene Bank accession Number** |
| --- | --- | --- | --- |
| TNFα [[1](#_ENREF_1)] | CCAGGCGGTGCCTATGTCT | GGCCATTTGGGAACTTCTCAT | NM_013693 |
| IL1β | TGAAGTTGACGGACCCCAAA | TGATGTGCTGCTGCGAGATT | NM_008361 |
| ICAM [[2](#_ENREF_2)] | CCCACGCTACCTCTGCTC | GATGGATACCTGAGCATCACC | NM_010493 |
| VCAM [[2](#_ENREF_2)] | TGGTGAAATGGAATCTGAACC | CCCAGATGGTGGTTTCCTT | NM_011693 |
| IL6 | AGTTGCCTTCTTGGGACTGA | TCCACGATTTCCCAGAGAAC | NM_031168 |
| tslp | TCCTATCCCTGGCTGCCCTTCA | TGTGCCATTTCCTGAGTACCGTCA | NM_21367 |
| Sprr2a [[3](#_ENREF_3)] | GAACCTGATTCTGAGACTCAA | GCACACTACAGGACGACAC | NM_11468 |
| Sprr2d | CTGGTACTCAAGGCCGAGAC | CAGGGCACTTTGGTGGAG | NM_011470 |
| Il4 | CATCGGCATTTTGAACGAG | CGAGCTCACTCTCTGTGGTG | NM_021283 |
| Il13 | CCTCTGACCCTTAAGGAGCTTAT | CGTTGCACAGGGGAGTCT | NM_008355 |
| Hprt [[1](#_ENREF_1)] | GTTGGATACAGGCCAGACTTTGTTG | GATTCAACTTGCGCTCATCTTAGGC | NM_013556 |
| Gadph [[1](#_ENREF_1)] | CTCATGACCACAGTCCATGC | CACATTGGGGGTAGGAACAC | NM_008084 |

**Table S3:** Real time PCR human primers used in this study

| **Target Gene** | **Forward** | **Reverse** | **Gene Bank accession Number** |
| --- | --- | --- | --- |
| IL1β | TACCTGTCCTGCGTGTTGAA | TCTTTGGGTAATTTTTGGGATCT | NM_000576 |
| TSLP | CCAGGCTATTCGGAAACTCA | TCCAGACATTTATTGGTTGTGACT | NM_033035.4 |
| IL13 | AGCCCTCAGGGAGCTCAT | TGATGCTCCATACCATGCTG | NM_002188.2 |
| SPRR2A | AACCCCTGGTACCTGAGCA | CTTGCACTGCTGTTGAT | NM_005988.2 |
| RPL13A | Qiagen, QuantiTect Primer Assays QT02321333 |  | NM_012423 |

**Table S4:** List of antibodies used in this study

| **Antibody** | **Company (provider)** | **Use** |
| --- | --- | --- |
| anti-keratin 5 | Prof. Daniel Hohl | IF |
| anti-keratin 6 | Covance #PRB-169P (Berkeley CA, USA) | IF |
| anti-corneodesmosin | Prof. Michel Simon [[4](#_ENREF_4)] | WB and IF |
| anti-desmocolin 1 | Dr. Peter Koch [[5](#_ENREF_5)] | WB and IF |
| anti-desmoglein 1+2, clone DG3.10 | Progen #61002, Germany | WB and IF |
| anti-SPRR2A and 2B | Adipogen #AG-25B-0002 , Switzerland | IF |
| anti-p50/p105 clone E381 | Millipore #04-234, Temecula CA, USA | WB |
| Anti-p50 H-119 | Santa Cruz # sc-7178, Switzerland | IF |
| anti-p-NFκB p65 (Ser 311) | Santa Cruz #sc-33039, Switzerland | WB |
| anti-phospho Stat3 (Tyr705), clone EP2147Y | Millipore #04-1059, Temecula CA, USA | WB |
| anti-actin | Sigma #A-2066, Switzerland | WB |

**Table S5:** Comparison of *a ma/ft ma/ft J* mouse characteristics with human AD and IV features

|  | ***a ma/ft ma/ft J* mouse** | **Human IV** | **Human AD** |
| --- | --- | --- | --- |
| FLG mutations | + | + | 50% |
| Dry, scaly skin | + | + | + |
| Loss of keratohyalin granules | F-granules absent | + | 50% |
| Granular layer | Attenuated | Attenuated or absent | According to FLG expression |
| Thickened sc | - | + | - |
| Acanthosis | + | - | + |
| Inflammatory infiltrates | + | - | + |
| IgEs | Yes (depending on the age and experimental setting) | - | + |
| TSLP | + | + | + |
| IL1β, NFκB signaling | + | - | + |
| Th2-cytokines | + | - | + |
| Sprr2 | + | - | + |

**Table S6:** Review on *a ma/ft ma/ft J* , *flg^ft^/flg^ft^* and *flg^-/-^* mouse main characteristics, according the literature. C57bkl/6J WT mice were used as controls in all studies.

| **Age**  **Mouse**  **model** | **E17.5** | **3-4 days** | **4-6 weeks** | **8 weeks** | **12 weeks** | **>20 weeks** |
| --- | --- | --- | --- | --- | --- | --- |
| ***a ma/ft ma/ft J*** | Barrier dysfunction (permeability)  Peridermal retention  (🡑 K6)  **(Okano et al., 2012)** | 🡑IL1β, TSLP, IL4, IL13,  🡑Sprr2a  **(our paper)**  = pH  =KLK5, KLK7 KLK14  **(Moniaga et al., 2013, our observation)**  🡑TSLP* **(Moniaga et al., 2013)**  *murine keratinocytes from neonates | Erythema,  scaling, 🡑TEWL  **(Moniaga**  **et al. 2010)** | hyperkeratosis, lymphocytic infiltration,  🡑IgE and IgG1, 🡑IL17, IL6, IL23, TSLP  = IL4, IL13, IFNγ  **(Moniaga et al., 20010; Oyoshi et al,. 2010)**  🡑pH  🡑KLK5, KLK7, KLK14  **(Moniaga et al., 2013)** | Barrier dysfunction, low inflammation  Impaired lamellar body secretion  **(Scharschmidt et al., 2009)** | Visible eczematous lesions  **(our observations, Oyoshi et al., 2010; Moniaga et al., 2010)** |
| **,*flg^ft^/flg^ft^*** |  | No obvious abnormalities  **(Fallon et al., 29009)**    🡑Il1β*, IlRA*  **(Kezic et al., 2012)**  *murine keratinocytes from neonates |  | Normal TEWL  Mild ortho-  keratotic hyper-  keratosis  acanthosis  Lymphocytic infiltrates  **(Fallon et al., 2009)**  High Il1β , IlRA, unchanged IL1α  **(Kezic et al., 2012)** |  |  |
| ***flg^-/-^***  ***(Kawasaki et al.2012)*** |  | Dry scaly skin, keratosis  =TEWL  =pH | Dry scaly  skin, keratosis  = TEWL  = pH | Dry scaly skin, keratosis  = TEWL  = pH  🡑permeability |  |  |

**SUPPORTING REFERENCES**

1. Roger T, Lugrin J, Le Roy D, Goy G, Mombelli M, et al. (2011) Histone deacetylase inhibitors impair innate immune responses to Toll-like receptor agonists and to infection. Blood 117: 1205-1217.

2. Toubas J, Beck S, Pageaud AL, Huby AC, Mael-Ainin M, et al. (2011) Alteration of connexin expression is an early signal for chronic kidney disease. Am J Physiol Renal Physiol 301: F24-32.

3. Yang J, Meyer M, Muller AK, Bohm F, Grose R, et al. (2010) Fibroblast growth factor receptors 1 and 2 in keratinocytes control the epidermal barrier and cutaneous homeostasis. The Journal of cell biology 188: 935-952.

4. Montezin M, Simon M, Guerrin M, Serre G (1997) Corneodesmosin, a corneodesmosome-specific basic protein, is expressed in the cornified epithelia of the pig, guinea pig, rat, and mouse. Exp Cell Res 231: 132-140.

5. Cheng X, Mihindukulasuriya K, Den Z, Kowalczyk AP, Calkins CC, et al. (2004) Assessment of splice variant-specific functions of desmocollin 1 in the skin. Molecular and cellular biology 24: 154-163.
